# Updating approach for lexicographic optimization-based planning to improve cervical cancer plan quality

**DOI:** 10.1007/s12672-023-00800-5

**Published:** 2023-09-30

**Authors:** Paolo Caricato, Sara Trivellato, Roberto Pellegrini, Gianluca Montanari, Martina Camilla Daniotti, Bianca Bordigoni, Valeria Faccenda, Denis Panizza, Sofia Meregalli, Elisa Bonetto, Peter Voet, Stefano Arcangeli, Elena De Ponti

**Affiliations:** 1 Medical Physics Department, Fondazione IRCCS San Gerardo Dei Tintori, Monza, Italy; 2 Department of Physics, University of Milan, Milan, Italy; 3 Medical Affairs, Elekta AB, Stockholm, Sweden; 4 Department of Physics, University of Milano Bicocca, Milan, Italy; 5 School of Medicine and Surgery, University of Milan Bicocca, Milan, Italy; 6 Department of Radiation Oncology, Fondazione IRCCS San Gerardo Dei Tintori, Monza, Italy; 7 Research Clinical Liaison, Elekta AB, Stockholm, Sweden

**Keywords:** Lexicographic optimization, Automated planning, Cervical cancer, VMAT, Plan quality, Plan comparison

## Abstract

**Background:**

To investigate the capability of a not-yet commercially available fully automated lexicographic optimization (LO) planning algorithm, called mCycle (Elekta AB, Stockholm, Sweden), to further improve the plan quality of an already-validated Wish List (WL) pushing on the organs-at-risk (OAR) sparing without compromising target coverage and plan delivery accuracy.

**Material and Methods:**

Twenty-four mono-institutional consecutive cervical cancer Volumetric-Modulated Arc Therapy (VMAT) plans delivered between November 2019 and April 2022 (50 Gy/25 fractions) have been retrospectively selected. In mCycle the LO planning algorithm was combined with the a-priori multi-criterial optimization (MCO). Two versions of WL have been defined to reproduce manual plans (WL01), and to improve the OAR sparing without affecting minimum target coverage and plan delivery accuracy (WL02). Robust WLs have been tuned using a subset of 4 randomly selected patients. The remaining plans have been automatically re-planned by using the designed WLs. Manual plans (MP) and mCycle plans (mCP01 and mCP02) were compared in terms of dose distributions, complexity, delivery accuracy, and clinical acceptability. Two senior physicians independently performed a blind clinical evaluation, ranking the three competing plans. Furthermore, a previous defined global quality index has been used to gather into a single score the plan quality evaluation.

**Results:**

The WL tweaking requests 5 and 3 working days for the WL01 and the WL02, respectively. The re-planning took in both cases 3 working days. mCP01 best performed in terms of target coverage (PTV V_95%_ (%): MP 98.0 [95.6–99.3], mCP01 99.2 [89.7–99.9], mCP02 96.9 [89.4–99.5]), while mCP02 showed a large OAR sparing improvement, especially in the rectum parameters (e.g., Rectum D_50%_ (Gy): MP 41.7 [30.2–47.0], mCP01 40.3 [31.4–45.8], mCP02 32.6 [26.9–42.6]). An increase in plan complexity has been registered in mCPs without affecting plan delivery accuracy. In the blind comparisons, all automated plans were considered clinically acceptable, and mCPs were preferred over MP in 90% of cases. Globally, automated plans registered a plan quality score at least comparable to MP.

**Conclusions:**

This study showed the flexibility of the Lexicographic approach in creating more demanding Wish Lists able to potentially minimize toxicities in RT plans.

**Supplementary Information:**

The online version contains supplementary material available at 10.1007/s12672-023-00800-5.

## Introduction

With the advent of inverse planning and adaptive techniques in all the domains of the Radiotherapy (RT) world from Hadrontherapy to Conventional RT and Brachytherapy, the need to automate workflows to ensure speed and consistency has become more and more pressing. In this framework, the evolution towards automated contouring and automated planning techniques has been perceived as the resolution of these two classical bottlenecks, always requiring extensive manual activities by radiation oncologists and planners.

Nowadays, automatic planning tools are widely known and spread with their main characteristic to emulate the human planners’ interactions with the treatment planning system (TPS). Their operation mode is largely described in the literature. It is so widely known how automation can reduce planning time, and increase plan efficiency and consistency, potentially leading to improved patient outcomes [1–7]. The automated-planning capability to generate plans at least comparable with the manual ones has been extensively reported [2, 8–15]. A question naturally arises: “If automated strategies can easily achieve clinical performances can we go further by stressing these techniques? And if the answer is ‘yes’, how far can we go?”. Only a few studies focus on automated tools updating and upgrading, deeply investigating their performances. Two studies recently reported how updating a Knowledge-Based Planning (KBP) model would improve plan quality and consistency, although overfitting issues have to be carefully managed [16, 17], and how developing different KBP models on the same plan library optimized by different TPS could lead to different dosimetric and modulation complexity performances [18]. The not-yet commercially available fully-automated lexicographic optimization (LO) planning algorithm, called mCycle (Elekta AB, Stockholm, Sweden) has been recently validated in head and neck volumetric-modulated arc therapy (VMAT) treatment planning [5], conventional treatment of prostate cancer, prostate Stereotactic Body Radiation Therapy (SBRT), rectal cancer [19], prostate treatment on an MR-Linac [6]. While the Erasmus MC Cancer Institute of Rotterdam first introduced and implemented LO in the self-standing iCycle software [13], now mCycle is newly implemented into the Monaco TPS research version (v5.59.13). The LO optimization problem follows the hierarchical order of a treatment site specific list of requests, a so-called Wish-List (WL). The WLs are generated by imitating the plan discussion process between radiation oncologists and planners and are characterized by clinical and planning constraints (CC and PC, respectively), which cannot be violated, and a list of prioritized objective functions according to their importance degree [20, 21]. At our Institute, a recent validation in cervical cancer treatment has been concluded, demonstrating that automated plans were dosimetric comparable with manual plans, but outperformed manual ones at the blinded clinical scoring [21]. Now that mCycle has been validated in different anatomic sites, the same question on further planning performances arises. The aim of this study is to deeply explore mCycle capability to go further the manual plan quality, stressing the organs-at-risk (OARs) sparing while preserving a minimum acceptable target coverage and accuracy of the plan delivery. The following comparison of these plans has been based on dose distributions, complexity, delivery accuracy, and clinical acceptability.

## Material and methods

### Pathology

Cervical cancer is one of the most common cancers in females worldwide for both incidence and mortality [22]. According to the Worldwide Health Organization (WHO), about 340,000 females die of cervical cancer every year in the world, 90% of deaths occur in low- and middle-income countries, and 99% of cervical cancers are caused by infection with human papillomavirus (HPV) [23]. Cervical cancer patients represent more than 10% of the overall annual workload at our Department of Radiation Oncology.

### Patient population

Twenty-four mono-institutional consecutive cervical cancer patients treated between November 2019 and April 2022 have been retrospectively selected. In order to be as generalizable as possible, 9 out of 24 patients had undergone surgery and the other 15 patients had not, thus challenging the mCycle algorithm’s robustness to manage very different anatomies. The criterion of inclusion was a prescription dose of 50 Gy in 25 fractions, representing the most frequent Institute’s cervical cancer protocol. On the other hand, the presence of mono- or bi-lateral femoral prosthesis was considered an exclusion criterion due to a non-standard planning setup chosen for each specific case. All patients underwent a CT simulation with a 3 mm slice thickness in the supine position. A specific OARs preparation requiring an empty rectum and a filled bladder was carried out before the simulation and each treatment fraction to ensure internal anatomy as reproducible as possible [24]. Two experienced radiation oncologists contoured the original structure sets that have been used for planning and analysis purposes. The structure sets included targets, involving cervix, uterus (if present), proximal vagina, pelvic nodes, and OARs, i.e., rectum, bladder, bowel bag (outer contour of bowel loops including the mesenterium, upper limit linked to the target extension, sigmoid as lower limit), and femoral heads [24]. The planning target volume (PTV) was defined as a 7-mm isotropic expansion of the clinical target volume (CTV) as prescribed by the institutional protocol. The selected DICOM sets were deeply anonymized by RSNA-CTP DicomAnonymizer (MIRC project, RSNA) prior to conducting the research. No ethical committee approval was needed for this retrospective dosimetric planning study.

### Manual treatment planning

Clinical manual VMAT plans (MP) were optimized according to Institutional protocol dose tolerances PTV V_95%_ > 97%, acceptable > 95%, D_1%_ < 107%; rectum D_50%_ < 44.7 Gy; bladder D_50%_ < 57.3 Gy; small bowel V_45Gy_ < 195 cm^3^; femoral heads D_5%_ < 44.7 Gy [25–28]. All plans were optimized with Monaco TPS (version 5.51.10) using a 6 MV-coplanar dual 330°-arc (165–195°) with up to 150 control points (CP), and sequencing parameters such as 1 cm-minimum segment width (SW), and highly smoothed fluence. The parameters of the Monte Carlo calculation were a 3 mm-dose grid and 1%-statistical uncertainty per plan. Patients were treated using an Elekta VersaHD linear accelerator equipped with the Agility Multileaf Collimator (MLC, 160 leaves, 5 mm thickness, up to 6.5 cm/sec), with the Monitor Unit (MU) calibration of 1 MU = 1 cGy with the reference field at the reference depth. The clinical objectives were accounted for in the a-priori MCO of the clinical Monaco^™^ TPS, as comprehensively treated described in Trivellato et al. [21]. Final normalization of dose distribution to achieve minimum PTV coverage or to satisfy small bowel constraints has been allowed. Whenever it was not possible to respect the above constraints for PTV or at least one OAR, minor or major deviations were discussed with and accepted by the approving clinician.

### mCycle auto-planning

Unlike the previous iCycle, mCycle is now implemented into the Monaco TPS research version (v5.59.13) and it applies the LO approach to the typical Monaco cost functions and Monte Carlo Algorithm (XVMC). Moreover, it is based on a completely new code including a new mathematical solver and a new patient model [19]. Furthermore, a new Segment optimization has been made available, the Pseudo-Gradient Descent Segment Shape Optimizer (PGDSSO). It is a new method of refining a set of MLC segments for a plan using a search method analogous to gradient descent. At each loop, segments are chosen starting from the desired maximum number of segments among all the possible segments and then gradually reduced by 10% each loop down to 50%, where the algorithm then stays throughout the rest of the optimization.

The mCycle fluence optimization (FMO) uses a two-pass automated lexicographic MCO in which constraints and prioritized objectives are managed by the planner through the WL. The WL tuning process is a multi-step iterative method described by Hussein et al. [2], while the description of the two-passed fluence LO was thoroughly discussed by Trivellato et al. [21].

The previous WL tweaking has been performed aiming to reproduce the manual plans (WL01). In this study, a second WL was generated to investigate the possibility to improve the plan quality in terms of organs-at-risk (OARs) sparing without affecting plan delivery accuracy. The WLs tuning has been done on the same subset of CTs and structure sets to get a robust hierarchical list of requests giving clinically acceptable dose distributions limiting any manual intervention as much as possible.

The designed WLs have been exploited to automatically re-plan the remaining selected treatment plans, using the same treatment arc with up to 150 CP, and the same sequencing parameters of the manual plans, highly smoothed fluence, 3 mm-dose grid, and 0.3%-statistical uncertainty per CP in the Monte Carlo calculation. No further WLs changes were allowed in this test phase. To satisfy the clinical objectives, the manual interventions on mCycle plans (mCP01 and mCP02, respectively) were limited to a re-optimization with a 0.75 cm-minimum segment width or a final re-normalization of the dose distribution in order to reach the minimum PTV coverage of V_47.5 Gy_ > 95% or to comply with the small bowel constraint V_45Gy_ < 195 cm^3^. These interventions were allowed to ensure comparability with other plans, similar to our manual planning workflow. Any other extensive manual tweaking has been avoided to prevent introducing any bias in the plan comparison.

### Plan comparison

MP, mCP01, and mCP02 were recalculated with a statistical uncertainty of 0.5% per plan to provide an unbiased comparison. Manual and automatic plans were compared by assessing differences in PTV V_100%_, V_95%_, and D_1%_. The dose distributions were compared in terms of the conformality index (CI_95%_ and CI_50%_), defined by the ratio between the total volume covered by the specified dose (95% and 50% of the prescription dose) and the volume of the PTV, and the homogeneity index (HI), represented by the formula HI = (D_2%_–D_98%_)/D_p_, where D_p_ is the prescription dose. The OAR mean doses, the rectum and bladder D_50%_, and the femoral heads D_5%_ have been also reported. The plan quality score introduced by Trivellato et al. [21] was used in the comparison.

### Plan complexity and delivery accuracy

Manual and automated planning modalities have also been analyzed in terms of plan complexity through the total number of MUs, the number of segments, and the modulation complexity score (MCS), as defined by McNiven [29]. All plans have been recalculated on the CT scan of the Delta^4+^ phantom (ScandiDos, Uppsala, Sweden) using a 2-mm grid and a 0.5%-statistical uncertainty. All plans were delivered at the linac VersaHD to test the plan delivery accuracy and to assess the agreement between calculated and measured dose distributions by performing a 3D-gamma analysis (ɣ). Automatic and manual plans were consecutively delivered on the phantom on the same day to avoid daily delivery variations. The local ɣ has been performed with Scandidos software (version 1.00.0180). The gamma passing rate was evaluated with a local 3%/3 mm criteria [PR(3%/3 mm)] excluding any pixel registering a dose lower than 8% of the maximum dose (threshold), according to the institutional clinical routine. A ɣ-passing criterion of 90% was used, as clinically applied [30].

### Blind physician scoring

To clinically evaluate the mCycle plans, two experienced radiation oncologists (ROs) have been asked to perform an independent blind plan evaluation. The request was to rank the three competing plans in order of acceptability as 1st, 2nd, and 3rd according to the institutional guidelines, i.e., based on dose distribution, dose-volume histograms, and clinical objectives. It’s worth noticing that the plans were randomly anonymized and no information about the planning method was provided. Cohen’s kappa coefficient has been calculated to assess the agreement between the two raters, providing valuable insights into the degree of concordance. Cohen’s kappa score was defined as excellent (k > 0.81), good (0.61 < k < 0.80), moderate (0.41 < k < 0.60), fair (0.21 < k < 40), and poor (k < 0.20) [31]. Moreover, the RO ranking has been evaluated in terms of the ranking agreement, which provides information about how many times the two raters ranked a plan in the same position, and the total agreement, as the sum of each ranking agreement.

### Statistical analysis

The normality test of Shapiro–Wilk has been performed to establish whether to perform the parametric t-test or the non-parametric Wilcoxon rank-sum test. The Bonferroni correction for multiple tests has been applied and the selected significance level has been set at 5% (p = 0.05). According to whether a sample is parametric or non-parametric, Bartlett’s or Levene’s test has been carried out to check if the samples belong to populations with equal variances [4]. The analysis of Bland–Altman (B-A) plots was used to compare two measurements of the same variables and to identify any systematic differences, outliers, and particular disagreement patterns [33]. Furthermore, the box-and-whisker plots were used to display in a single chart how data of different populations are distributed. All the statistical tests have been performed using Rstudio (2021.09.0), while the B-A plots have been performed using Python 3-Release (Python 3.9.7).

## Results

### Wish-Lists tweaking

The WL01 and WL02 preparation and fine tuning required about 5 and 3 working days, respectively. The WL01 was the starting point of WL02 tuning: WL01 has been iteratively modified to reach the WL02 goals of getting OARs sparing as high as possible, accepting a slightly lower target coverage without compromising the plan delivery accuracy. The two detailed WLs are presented in Tables 1 and 2. In both cases, the fulfilment of the bowel bag constraint is indicated as CC because its violation implies plan rejection most of the time. It is followed by dose gradient requests (PC). The main differences between the two WLs regard the PTV coverage and the OAR mean doses requests priority order. In the WL02, a less strict PTV coverage request is performed as a 1^st^-priority request, and a last-priority level request was added to achieve PTV coverage as high as possible, while the bladder and rectum mean doses claims are swapped with the right- and left-femoral heads ones. In both WLs, if there was a double PTV with the same dose prescription (PTV uterus and PTV pelvis) the requests were doubled and kept both as first-priority objectives function.

**Table 1:** mCycle Wish-List 01 for auto-planning of cervical cancer at 50 Gy in 25 fractions

Wish-List 01			
Structure	Cost function (parameter)	Margin (cm)	Limit
Clinical constraints			
PTV	Quadratic overdose (52 Gy)		< 0.02 Gy
Bowel bag	Overdose DVH (45 Gy)		< 195.0 cm^3^
External	Maximum Dose		53.4 Gy
Planning constraints			
External	Quadratic overdose (50 Gy)	0.0	< 0.1 Gy
External	Quadratic overdose (45 Gy)	0.3	< 0.2 Gy
External	Quadratic overdose (40 Gy)	0.6	< 0.2 Gy
External	Quadratic overdose (35 Gy)	0.9	< 0.2 Gy
External	Quadratic overdose (25 Gy)	2.5	< 0.3 Gy

Objectives				
Priority	Structure	Cost function (parameter values)	Margin (cm)	Goal value (sufficient)
1	PTV	Target EUD (0.5)		50.0 Gy
1	PTV	Target Penalty (99%)		50.0 Gy
2	Rectum	Parallel (40 Gy, k = 3)	0.3	< 30.0%
2	Bladder	Parallel (40 Gy, k = 3)	0.3	< 33.5%
3	Rectum	Serial (k = 15)		< 46.0 Gy
3	Bowel bag	Parallel (40 Gy, k = 3)	0.3	< 20.0%
3	Bowel bag	Serial (k = 15)		< 43.0 Gy
3	Bladder	Serial (k = 15)		< 47.0 Gy
4	Bowel bag	Overdose DVH (45 Gy)		< 14.0% (7.0%)
4	Right femoral head	Serial (k = 15)		< 38.0 Gy
4	Left femoral head	Serial (k = 15)		< 38.0 Gy
5	External	Conformality		< 0.75
6	Right femoral head	Serial (k = 1)		< 30.0 Gy
6	Left femoral head	Serial (k = 1)		< 30.0 Gy
7	Rectum	Serial (k = 1)		< 30.0 Gy
8	Bladder	Serial (k = 1)		< 35.0 Gy

Priority: order list according to which the objectives (cost functions) are optimized. Margin: creates a buffer zone between the PTV and overlapping structures to avoid conflict between the applied cost functions of each structure

*PTV* planning target volume, *DVH* dose volume histogram, *EUD* equivalent uniform dose

**Table 2:** mCycle Wish-List 02 for auto-planning of cervical cancer at 50 Gy in 25 fractions

Wish-List 02			
Structure	Cost function (parameter)	Margin (cm)	Limit
Clinical constraints			
PTV	Quadratic overdose (52.5 Gy)		< 0.02 Gy
Bowel bag	Overdose DVH (45 Gy)		< 195.0 cm^3^
External	Maximum dose		53.5 Gy
Planning constraints			
External	Quadratic overdose (50 Gy)	0.0	< 0.1 Gy
External	Quadratic overdose (45 Gy)	0.3	< 0.2 Gy
External	Quadratic overdose (40 Gy)	0.6	< 0.2 Gy
External	Quadratic overdose (35 Gy)	0.9	< 0.2 Gy
External	Quadratic overdose (25 Gy)	2.5	< 0.3 Gy

Objectives				
Priority	Structure	Cost function (parameter values)	Margin (cm)	Goal value (sufficient)
1	PTV	Target Penalty (95%)		47.5 Gy
1	PTV	Target Penalty (50%)		50.0 Gy
2	Rectum	Parallel (40 Gy, k = 3)		< 30.0%
2	Bladder	Parallel (40 Gy, k = 3)		< 40.0%
3	Rectum	Serial (k = 15)		< 46.0 Gy
3	Bowel bag	Parallel (40 Gy, k = 2)		< 20.0%
3	Bladder	Serial (k = 20)		< 47.5 Gy
4	Bowel bag	Overdose DVH (45 Gy)		< 14.0% (7.0%)
4	Right femoral head	Serial (k = 15)		< 38.0 Gy
4	Left femoral head	Serial (k = 15)		< 38.0 Gy
5	External	Conformality		< 0.75
6	Rectum	Serial (k = 1)		< 30.0 Gy (34.5%)
7	Bladder	Serial (k = 1)		< 35.0 Gy
8	Left femoral head	Serial (k = 1)		< 30.0 Gy
8	Right femoral head	Serial (k = 1)		< 30.0 Gy
9	PTV	Underdose DVH (47.5 Gy)		> 99.0%

Priority: order list according to which the objectives (cost functions) are optimized. Margin: creates a buffer zone between the PTV and overlapping structures to avoid conflict between the applied cost functions of each structure

*PTV* planning target volume, *DVH* dose volume histogram, *EUD* equivalent uniform dose

### mCycle auto-planning

The automatic re-planning for the remaining 20 patients (test set) took 3 working days for each WLs. The obtained mCP01 and mCP02 required manual fine-tuning in 30% and 35% of plans, respectively. The plan re-normalization was required for 6 (30%) and 7 (35%) plans, respectively, while a re-optimization with 0.75 cm of minimum-SW was performed for 2 (10%) mCP01 and 1 (5%) mCP02.

### Dosimetric comparison

The median PTV volume was 1073.7 cm^3^ [608.4–1453.9 cm^3^].

**Fig. 1:**
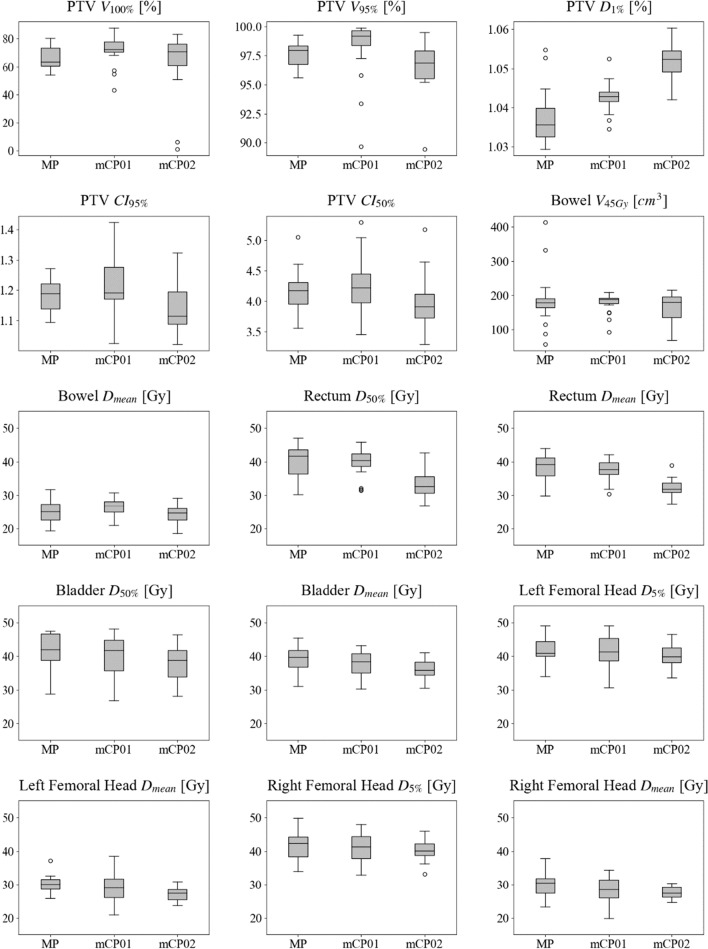
Box-and-whisker plots for PTV and OARs parameters for manual plans (MP) and mCycle plans (mCP01 and mCP02). Abbreviations: PTV: planning target volume V_#_: volume receiving more than # Gy, D_#_: dose received by the # % of contoured volume, D_mean_: mean dose, CI_#_: conformality index of the # dose

**Fig. 2:**
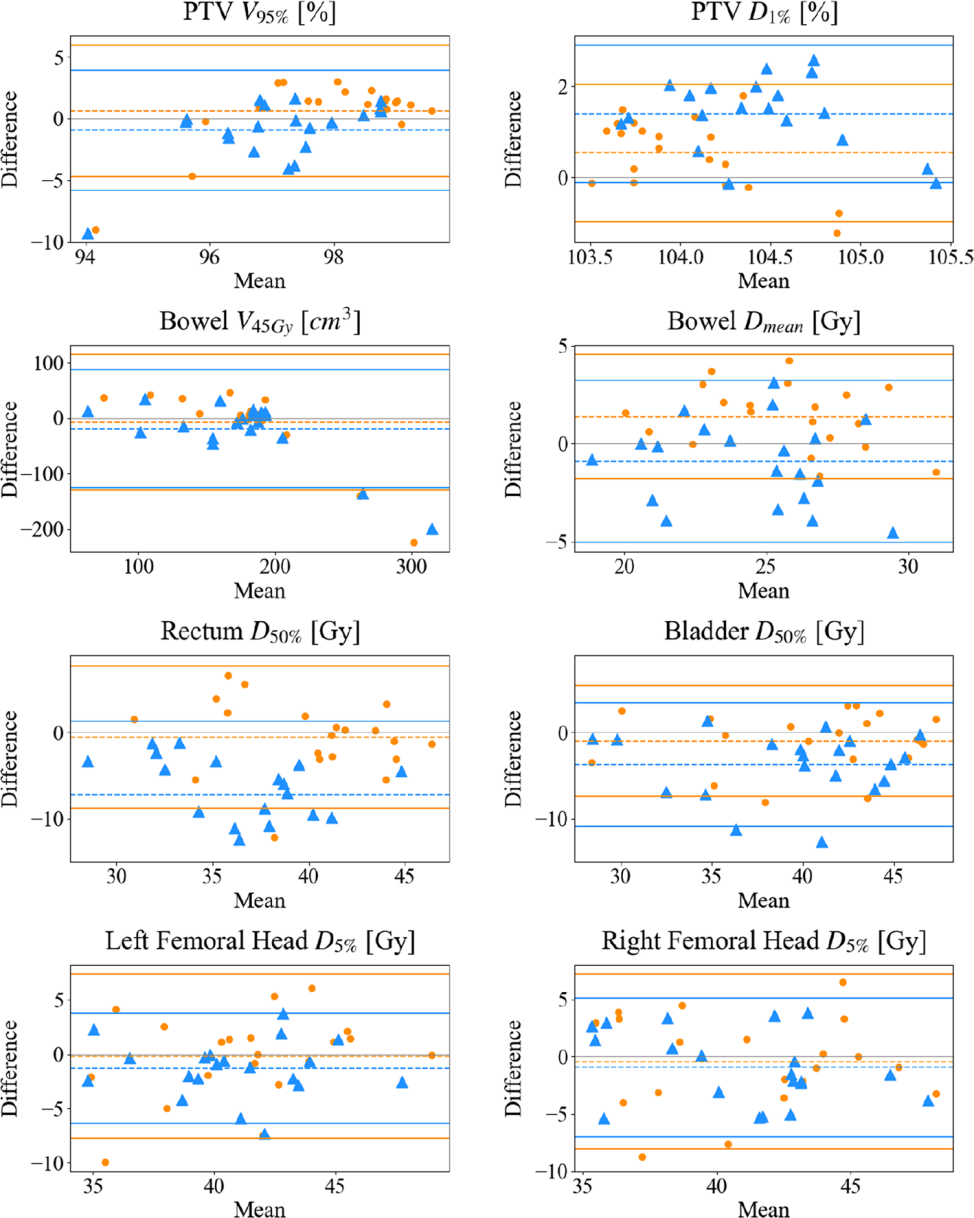
Bland Altman plots for PTV and OARs parameters for manual plans (MP) and mCycle plans (mCP01 and mCP02). In Bland Altman plots orange circles and blue triangles represent "MP vs mCP01" and “MP vs mCP02” comparison, respectively. Dashed lines: bias line, solid lines: agreement limits lines. Abbreviations: PTV: planning target volume, V_#_: volume receiving more than # Gy, D_#_: dose received by the # % of contoured volume, D_mean_: mean dose

Target dose results are summarized in Table 3. Although not statistically significant once the Bonferroni correction was applied, mCP01 showed a higher target coverage than MP. This coverage increase resulted statistically significant with respect to mCP02. This analysis demonstrated significant growth in the PTV D_1%_, never exceeding the protocol constraint. As it may be noticed in the related box-and-whisker and Bland Altman plots reported in Figs. 1 and 2 the data variability is less for AP than MP and the median value is generally higher for AP. Unlike the comparable conformality results, mCP02 is significantly more inhomogeneous than MP and mCP01.

**Table 3:** Comparison of original manual plans (MP) and mCycle plans (mCP01 and mCP02) in terms of PTV dose metrics

DOSE METRICS PTV		Median [range]	mCP01		mCP02	
			MVA p-values	VVA p-values	MVA p-values	VVA p-values
V_100%_ (%)^(2)^	MP	63.3 [54.2–80.4]	**0.040**/1.000	0.597	0.289/1.000	0.304
	mCP01	72.4 [43.3–87.7]			0.355/1.000	0.740
	mCP02	70.8 [50.9–83.0]				
V_95%_ (%)^(2)^	MP	98.0 [95.6–99.3]	**0.004**/0.120	0.391	0.094/1.000	0.103
	mCP01	99.2 [89.7–99.9]			**0.001/0.030**	0.803
	mCP02	96.9 [89.4–99.5]				
D_1%_ (%)^(2)^	MP	103.6 [102.9–105.5]	**0.001/0.030**	0.066	** < 0.001/ < 0.001**	0.490
	mCP01	104.3 [103.5–105.3]			** < 0.001/ < 0.001**	0.130
	mCP02	105.2 [104.2–106.0]				
CI_95%_^(1)^	MP	1.2 [1.1–1.3]	0.218/1.000	**0.018**	0.109/1.000	0.070
	mCP01	1.2 [1.0–1.4]			**0.006**/0.254	0.562
	mCP02	1.1 [1.0–1.3]				
CI_50%_^(1)^	MP	4.2 [3.6–5.1]	0.379/1.000	0.376	0.222/1.000	0.426
	mCP01	4.2 [3.5–5.3]			0.038/1.000	0.928
	mCP02	3.9 [3.3–5.2]				
HI^(2)^	MP	0.084 [0.068–0.120]	0.123/1.000	0.469	** < 0.001/0.004**	0.579
	mCP01	0.078 [0.061–0.130]			** < 0.001/0.012**	0.778
	mCP02	0.106 [0.082–0.145]				

Median values and ranges are reported

Bold: statistical significance (p < 0.05)

*MVA* median value analysis, *I* variance value analysis, *PTV* planning target volume, *V*_#_ volume receiving more than # Gy, *D*_#_ dose received by the # % of contoured volume, *CI*_#_ conformality index of the #% of the prescription dose

^(1)^Gaussian distribution

^(2)^not normal distribution

Non-corrected and Bonferroni-corrected p-values are reported (p value/corrected p value)

OAR results are reported in Table 4 and Figs. 1 and 2. While OAR sparing in mCP01 is comparable to MP, OAR metrics showed a slight if not large decrease in mCP02, with a statistical and clinical relevance in median values of the rectum D_50%_ and D_mean_. In B-A plots, it is worth noticing the lower position of mCP02 bias lines, meaning a mCP02 overall trend to overperform MP and mCP01. The variance test showed a statically relevant difference for the rectum D_mean_ and right femoral head D_mean_ parameters. In Fig. 1, it is worth noticing an extremely narrow boxplot for bowel V_45Gy_ coupled with a cluster of points in the B-A plot just below the constraint (Fig. 2).

**Table 4:** Comparison of original manual plans (MP) and mCycle plans (mCP01 and mCP02) in terms of OARs dose metrics

DOSE METRICS OARs		Median [range]	mCP01		mCP02	
			MVA p-values	VVA p-values	MVA p-values	VVA p-values
Bowel						
V_45 Gy_ (cm^3^)^(2)^	MP	179.2 [56.5 – 414.0]	0.344/1.000	0.080	0.862/1.000	0.371
	mCP01	188.3 [92.6 – 209.0]			0.543/1.000	0.147
	mCP02	180.4 [69.3 – 215.4]				
D_mean_ (Gy)^(1)^	MP	25.0 [19.3 – 31.7]	0.142/1.000	0.518	0.341/1.000	0.671
	mCP01	26.7 [20.8 – 30.8]			**0.018**/0.788	0.825
	mCP02	24.7 [18.5 – 29.1]				
Rectum						
D_50%_ (%)^(1)^	MP	41.7 [30.2 – 47.0]	0.713/1.000	0.430	** < 0.001/ < 0.001**	0.161
	mCP01	40.3 [31.4 – 45.8]			** < 0.001/ < 0.001**	0.534
	mCP02	32.6 [26.9 – 42.6]				
D_mean_ (Gy)^(1)^	MP	39.1 [29.7 – 44.0]	0.404/1.000	0.273	** < 0.001/ < 0.001**	**0.033**
	mCP01	37.7 [30.4 – 42.1]			** < 0.001/ < 0.001**	0.286
	mCP02	31.8 [27.4 – 38.9]				
Bladder						
D_50%_ (Gy)^(2)^	MP	42.0 [28.8 – 47.4]	0.583/1.000	0.631	**0.026**/0.780	0.914
	mCP01	41.6 [26.7 – 48.1]			0.081/1.000	0.698
	mCP02	38.8 [28.1 – 46.3]				
D_mean_ (Gy)^(1)^	MP	39.7 [30.9 – 45.4]	0.293/1.000	0.661	**0.005**/0.221	0.282
	mCP01	38.4 [30.3 – 43.1]			0.067/1.000	0.521
	mCP02	35.8 [30.5 – 40.9]				
Left femoral head						
D_5%_ (Gy)^(1)^	MP	40.9 [33.9 – 49.0]	0.872/1.000	0.239	0.300/1.000	0.907
	mCP01	41.3 [30.5 – 48.9]			0.380/1.000	0.197
	mCP02	39.7 [33.6 – 46.5]				
D_mean_ (Gy)^(1)^	MP	30.1 [25.8 – 37.2]	0.186/1.000	0.060	**0.005**/0.241	0.328
	mCP01	29.0 [20.9 – 38.4]			0.125/1.000	**0.005**
	mCP02	27.4 [23.6 – 30.9]				
Right femoral head						
D_5%_ (Gy)^(1)^	MP	42.3 [34.0 – 49.8]	0.734/1.000	0.973	0.468/1.000	0.264
	mCP01	41.3 [32.8 – 47.9]			0.699/1.000	0.278
	mCP02	40.0 [33.1 – 46.0]				
D_mean_ (Gy)^(1)^	MP	30.5 [23.2 – 37.8]	0.126/1.000	0.416	**0.034**/1.000	**0.014**
	mCP01	28.4 [19.8 – 34.3]			0.538/1.000	**0.001**
	mCP02	27.4 [24.6 – 30.3]				

Median values and ranges are reported

Bold: statistical significance (p < 0.05)

*MVA* median value analysis, *VVA* variance value analysis, *V*_#_ volume receiving more than # Gy, *D*_#_ dose received by the # % of contoured volume, *D*_*mean*_ mean dose

^(1)^Gaussian distribution

^(2)^not normal distribution

Non-corrected and Bonferroni-corrected p-values are reported (p value/corrected p value)

**Fig. 3:**
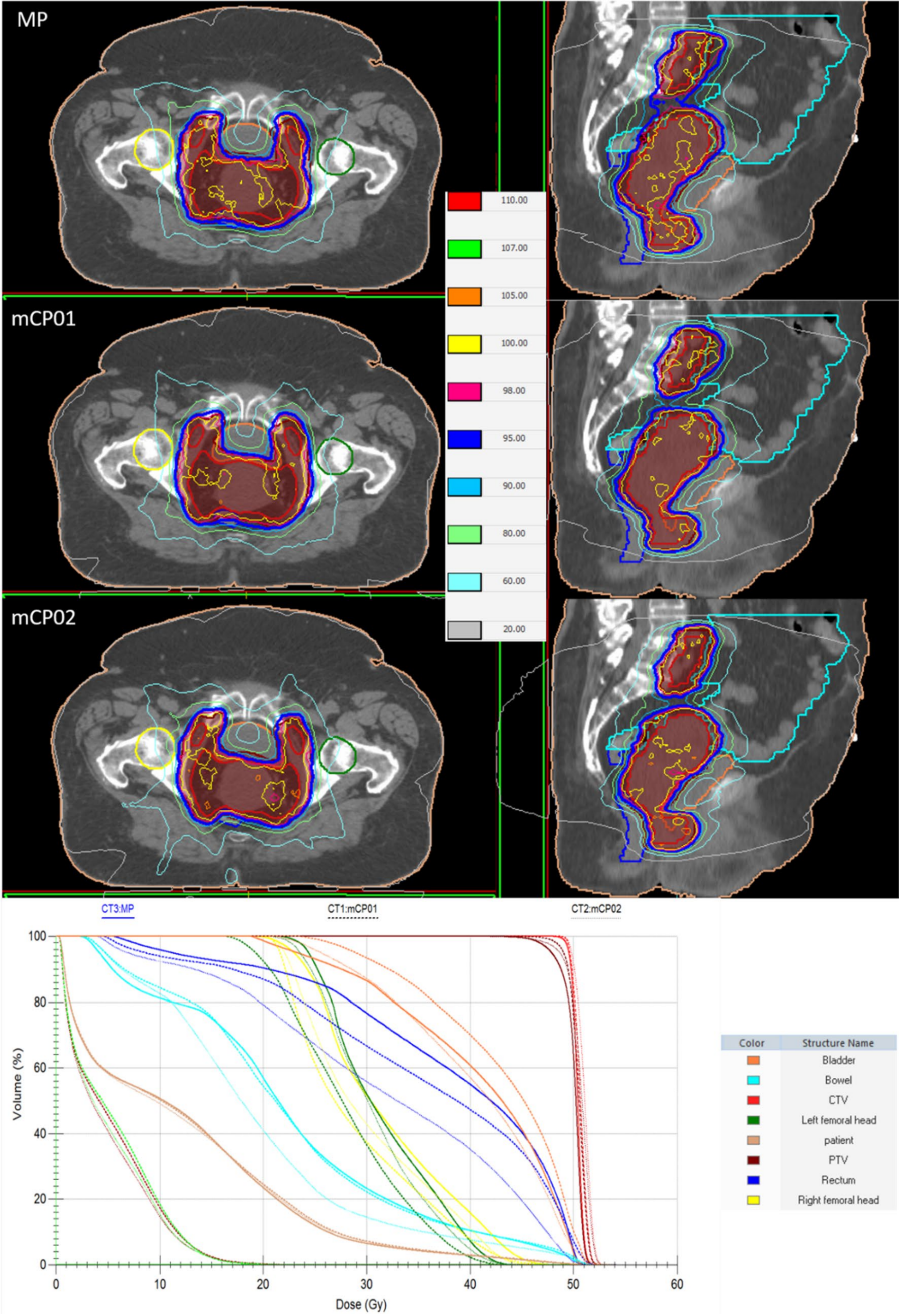
Dose distribution comparison of a manual plan (MP), a mCycle plan 01 (mCP01) and of a mCycle plan 02 (mCP02). The isodose color legend is reported in the middle while the contoured structures are CTV (red), PTV (brown), rectum (blu), bladder (orange), bowel (cyan), right femoral head (yellow), left femoral head (green), patient (pink). The DVH curves are reported as solid lines for MP, dashed lines for mCP01 and dotted lines for mCP02

The dose distributions and the relative DVHs for a representative patient are graphically reported in Fig. 3 illustrating the best performances of mCP01 regarding the PTV coverage and a large reduction in rectum and bladder doses in mCP02. Furthermore, it is worth noticing that mCP01 presented a slightly worse bladder DVH, as well as a larger extension of low doses with respect to MP and mCP02.

The results of the plan quality index (PQI) and its sub-metrics are reported in the Additional file 1. It is worth observing the PQI trend of mCP01 and mCP02 in comparison to the gold standard MP: the former shows a slight improvement in the overall plan quality, while the latter demonstrates comparable results.

### Plan complexity and delivery accuracy

**Fig. 4:**
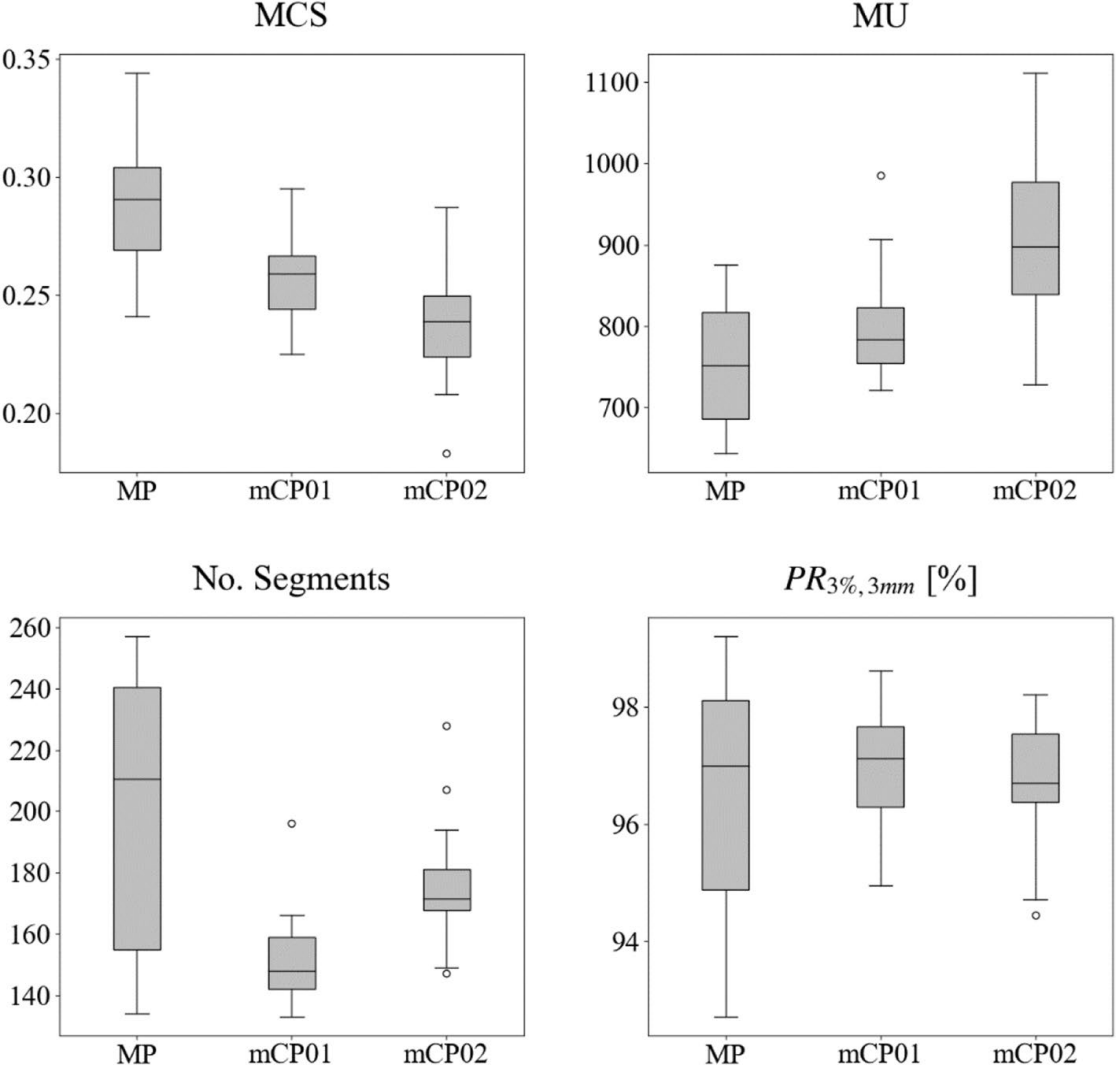
Box-and-whisker plots for plan complexity and delivery accuracy parameters for manual plans (MP) and mCycle plans (mCP01 and mCP02). Abbreviations: MCS: modulation complexity score, MU: monitor unit, PR: gamma passing ratio

**Fig. 5:**
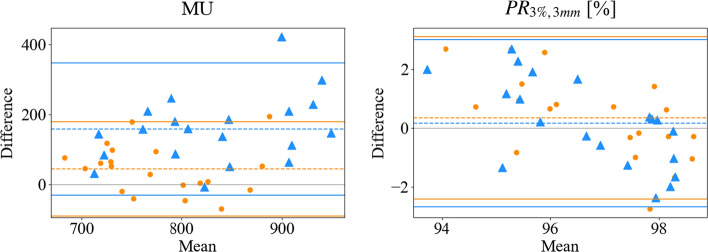
Bland Altman plots for plan complexity and delivery accuracy parameters for manual plans (MP) and mCycle plans (mCP01 and mCP02). In Bland Altman plots orange circles and blue triangles represent "MP vs mCP01" and “MP vs mCP02” comparison, respectively. Dashed lines: bias line, solid lines: agreement limits lines. Abbreviations: MU: monitor unit, PR: gamma passing ratio

Plan complexity and delivery accuracy results are reported in Table 5 and in Figs. 4 and 5. All metrics reported an increase in the complexity of the automated plans without affecting the accuracy of the plan delivery. MCS results showed an increased complexity passing from MP to mCP01 and from mCP01 to mCP02. This is coupled with an increase in the number of MU in mCP01 (6.9% [−7.93 ± 27.2]%, p = 1.000) and in mCP02 (22.5% [−0.74 ± 61.5]%, p = 0.005). This trend is clearly highlighted by the related bias line in Fig. 5. The lower number of segments in automatic plans was obtained thanks to the novel pseudo-gradient descent segment shape optimizer (PGDSSO). Comparing mCP02 to mCP01, a statistically significant increase is registered in the number of segments testifying the stronger request for plan modulation. As reported in Fig. 5 and Table 5 all the plan delivery accuracy metrics registered similar results, although PR (3%/3 mm) revealed a downward trend as the passing rate means an increase in BA plots. At the variance test, it is worth noticing that both mCP01 and mCP02 showed a statistically significant difference compared to MP due to higher minimum values of gamma passing rates.

**Table 5:** Comparison of original manual plans (MP) and mCycle plans (mCP01 and mCP02) in terms of plan complexity and plan delivery accuracy

PLAN complexity and delivery accuracy		Median [range]	mCP01		mCP02	
			MVA p-values	VVA p-values	MVA p-values	VVA p-values
MCS^(1)^	MP	0.29 [0.24–0.34]	** < 0.001/0.002**	**0.047**	** < 0.001/ < 0.001**	0.832
	mCP01	0.26 [0.23–0.30]			**0.010**/0.459	0.074
	mCP02	0.24 [0.18–0.29]				
MUs^(2)^	MP	751.2 [644.1–875.2]	0.086/1.000	0.188	** < 0.001/ < 0.001**	0.271
	mCP01	783.5 [721.2–985.1]			** < 0.001/0.005**	**0.049**
	mCP02	897.6 [728.5–1110.9]				
No. Segments^(2)^	MP	211 [134–257]	**0.001/0.030**	** < 0.001**	0.203/1.000	** < 0.001**
	mCP01	148 [133–196]			** < 0.001/ < 0.001**	0.599
	mCP02	172 [147–228]				
PR(3%/3 mm) (%)^(1)^	MP	97.0 [92.7–99.2]	0.441/1.000	**0.018**	0.712/1.000	**0.009**
	mCP01	97.2 [95.0–98.6]			0.687/1.000	0.794
	mCP02	96.7 [94.4–98.2]				

Median values and ranges are reported

Bold: statistical significance (p < 0.05)

*MVA* median value analysis, *VVA* variance value analysis, *MCS* modulation complexity score, *MU* monitor units, *PR* gamma passing rate

^(1)^Gaussian distribution

^(2)^not normal distribution

Non-corrected and Bonferroni-corrected p-values are reported (p value/corrected p value)

### Blind physician scoring results

All MP and mCPs were considered clinically acceptable. However, it has been highlighted that two MP presented a large deviation from the protocol criteria due to an overcoming of the bowel V_45Gy_ constraint strictly due to unfavorable anatomies. Despite these isolated cases, the remaining 58 plans satisfied the Institute protocol, although in a few cases minor deviations were accepted in PTV coverage, bowel, and femoral heads constraints.

It is crucial to consider the decision-making process of the ROs (Table 6). The two clinicians ranked mCP02 as the best strategy in 80% and 70% of cases, respectively, demonstrating its consistent performance. On the other hand, mCP01 was the preferred choice in 15% of cases. MPs were considered the best plan in merely 5% and 15% of cases, respectively, suggesting they were less favored by the ROs. The physicians’ total agreement was 63.3%, with a Cohen's kappa statistic of 0.45, indicating a moderate agreement among the raters.

**Table 6:** Plan ranking by two experienced radiation oncologists (RO1/RO2) of original manual plans (MP) and mCycle plans (mCP01 and mCP02)

Blind choice	Ranking			Ranking agreement	Total agreement
	1st	2nd	3rd		
MP	1/3 (5%/15%)	9/8 (45%/40%)	10/9 (50%/45%)	13 out of 20 (65%)	38 out of 60 (63.3%)
mCP01	3/3 (15%/15%)	8/7 (40%/35%)	9/10 (45%/40%)	13 out of 20 (65%)	
mCP02	16/14 (80%/70%)	3/5 (15%/25%)	1/1 (5%/5%)	12 out of 20 (60%)	

## Discussions

To our knowledge, this is the first study proving how it is possible to make a step further in the mCycle automatic planning for cervical cancer treatment. The results presented here confirmed how fast the automatic re-planning can be: for both WLs, the fast automatic re-planning took slightly more than one hour per plan to achieve 20 clinically acceptable and deliverable plans, supporting the idea that mCycle application in the clinical routine would strongly reduce planners’ workload on cervical treatment planning confirming what was proved for Erasmus-iCycle tool [34–36]. Furthermore, mCycle mostly created an optimal plan in an almost “one-button click” procedure without any planner intervention for manual tuning. A plan re-optimization with manual refinements was required in 10% and 5% of the cases for mCP01 and mCP02, respectively. This decrease in manual intervention can be seen as a further improvement of the WL leading to a further reduction of manual workload.

This study shows how auto-planning can generate at least comparable plans to manual-planning with higher efficiency and less inter-planner variability. It is worth noticing that these results did not affect what was already obtained in mCP01, especially looking at the sparing of the bowel proving how robust the LO is. Further studies are needed to evaluate the therapeutic ratio of mCP02 with respect to MP and mCP01, to do so these dosimetric results should be used to assess tumor control probability (TCP) and normal tissue complication probability (NTCP) for a different clinical endpoint.

The plan complexity analysis revealed the significantly higher complexity of mCycle plans compared to manual ones. Although the new PGDSSO led to a lower number of segments, the results showed the required MUs increase in the automatic plans. The WL02 pressing requests on OAR sparing led to a further complexity increase in the mCP02. Nevertheless, the preserved gamma passing ratio testified that the increased complexity did not affect the plan delivery accuracy, guaranteeing a treatment at least as safe and precise as the manual ones. This outcome seems to be common in several automatic planning systems. Bijman et al. demonstrated a slight increase of needed MUs for the mCycle system with mean differences between 11 and 19% linked to the anatomical site under consideration [19]. In the study by Heijmen et al., a median increase of 13% in the requested MUs obtained with the iCycle system was related to a larger reduction in rectum parameters [3]. Also Pinnacle Autoplanning and Genetic Planning Solution (Raystation TPS) showed statistically significant growth of the MUs per plan in all the explored anatomic sites without a lower passing rate in the pre–treatment verifications [4, 37]. On the other hand, Yang et al. demonstrated that RayStation TPS, coupled with the IronPython language platform, obtained a comparable number of MUs between automatic and manual plans for nasopharyngeal carcinoma, with at least comparable plan quality [38].

The blind choice performed by two experienced ROs revealed that a large decrease in OAR doses with a guaranteed minimal acceptable target coverage (mCP02) was mostly preferred to the higher target coverage of the opposing MP and mCP01. It is worth noticing that the blind choice resulted in a ‘moderate agreement’ in the final ranking which has been interpreted as mainly due to the selected 3-degrees scale of preferences permitting a full spectrum of plan discussion and acceptance levels. In particular, two interesting outliers (Figs. 1 and 2) have been comprehensively discussed because of a large decrease of the bowel V45Gy in the automated plans coupled with a strongly reduced PTV coverage. ROs finally and independently claimed that, given the possibility to choose between these treatments, they would have confirmed their choice in the clinical routine.

On the other hand, the PQI analysis showed that the two automated strategies are at least comparable to manual planning, with mCP01 slightly outperforming mCP02. It is worth noticing that ROs and PQI plan scoring disagreed in the mCP01 and mCP02 ranks. A possible explanation can be found in the relation between the PQI definition and mCP02 excellent results. mCycle capability to strongly reduce OARs parameters could change what ROs can expect. The PQI definition based on MP daily routine needs to be updated in light of mCycle capabilities, changing the sub-metrics weights to better fit the clinical evaluation. It has been demonstrated that automated strategies can be stressed to go further than the well-known manual planning routine. Furthermore, the possibility to generate different WLs allows to promptly answer clinicians’ requests: it would be possible to choose, patient by patient, the preferred compromise between DVH and plan complexity. Furthermore, these fast and customizable results suggest exploiting automated planning systems in a fast adaptive workflow soon, as demonstrated by Castriconi et al. [39] who reported that a well-defined KBP model could reduce planning time and inter-planner variability.

Only a few other studies faced the same issue in KBP planning. Hundvin et al. reported modest but significant improvements in both plan quality and consistency for high-risk prostate cancers performing a KBP model tuning [17], while Nakamura et al. showed that the last update of their model could make a better estimation of the DVH in the open-loop validation plans [16].

This study demonstrated how far an automated tool could lead the radiotherapy routine but it is worth emphasizing that the WLs development and evaluation is a challenging iterative process, strongly dependent on many factors as the institutional protocol on which it is based, the user know-how [35], and the lack of human and time resources to deeper investigate the tool full potential.

Future studies will focus on LO capabilities to adapt the here presented WLs to a multiple dose levels scenario, doubling and differentiating the PTVs requests and coherently adapting the OAR objective functions [5, 6]. Furthermore, a prospective analysis on a larger patient cohort is suggested. Indeed, Fogliata et al. highlighted those systematic investigations are needed to test the performance and robustness of the automated tools [9], and Wortel et al. pointed out the importance of periodically checking the quality and the acceptance rates of automatic plans after their clinical introduction [40]. Finally, to assess the generalization of the WLs, a multi-centric validation would be suggested.

## Conclusion

This comprehensive dosimetric and clinical study demonstrated that mCycle generates plans at least comparable and often superior to accepted manual plans in the selected patients’ cohort, outperforming manual plans at the blinded clinical ranking. The WL02 tuning showed the possibility of going further than manual planning quality in cervical cancer treatment. By considering the workload, dosimetric, and clinical advantages, mCycle proved to be an effective and flexible tool to generate automatic high-quality VMAT treatment plans according to the cervical treatment institutional protocol and its results are suggestive of a reliable methodology application to the clinical routine as soon as it will become commercially available.

### Supplementary Information

**Additional file 1: Table S7.** Comparison of original manual plans (MP) and mCycle plans (mCP01 and mCP02) in terms of Plan Quality Index (PQI) and its submetrics: coverage, OAR sparing, plan delivery accuracy, and plan complexity. Median values and ranges are reported. **Figure S6.** Box-and-whisker plots (left) and related Bland Altman plots (right) for PQI for manual plans (MP) and mCycle plans (mCP01 and mCP02). In Bland Altman plots orange circles and blue triangles represent "MP vs mCP01" and “MP vs mCP02” comparison, respectively. Dashed lines: bias line, solid lines: agreement limits lines. Abbreviations: PQI: Plan Quality Index.

## Data Availability

Research data are stored in an institutional repository and will be shared upon request to the corresponding author.

## Abbreviations

LO: Lexicographic optimization
OAR: Organ-at-risk
VMAT: Volumetric-modulated arc therapy
MCO: Multicriterial optimization
WL: Wish list
MP: Manual plans
mCP: MCycle plans
PTV: Planning target volume
RT: Radiotherapy
TPS: Treatment planning system
KBP: Knowledge-based planning
SBRT: Stereotactic body radiation therapy
CC: Clinical constraint
PC: Planning constraint
HPV: Human papillomavirus
CTV: Clinical target volume
CP: Control points
SW: Segment width
MLC: Multileaf collimator
MUs: Monitor units
FMO: Fluence matrix optimization
CI: Conformality index
MCS: Modulation complexity score
ROs: Radiation oncologists
PQI: Plan quality index
B-A: Bland–Altman
PGDSSO: Pseudo-gradient descent segment shape optimizer
TCP: Tumor control probability
NTCP: Normal tissue complication probability
