# Supplementary material for: Updating approach for lexicographic optimization-based planning to improve cervical cancer plan quality
Source: Discov Oncol. 2023 Sep 30;14:180. doi: 10.1007/s12672-023-00800-5 (PMC10541351; doi:10.1007/s12672-023-00800-5)
Supplement: Supplementary file 1 — Additional file 1: Table S7. Comparison of original manual plans (MP) and mCycle plans (mCP01 and mCP02) in terms of Plan Quality Index (PQI) and its submetrics: coverage, OAR sparing, plan delivery accuracy, and plan complexity. Median values and ranges are reported. Figure S6. Box-and-whisker plots (left) and related Bland Altman plots (right) for PQI for manual plans (MP) and mCycle plans (mCP01 and mCP02). In Bland Altman plots orange circles and blue triangles represent "MP vs mCP01" and “MP vs mCP02” comparison, respectively. Dashed lines: bias line, solid lines: agreement limits lines. Abbreviations: PQI: Plan Quality Index. [file 12672_2023_800_MOESM1_ESM.docx]

### Supplementary Material

**Plan quality score**

A Plan Quality Index (PQI) has been introduced to gather into a single score the quality judgment expressed by the Institute’s clinical team based on its knowledge and experience. This concept was first introduced by Nelms with the aim to remove any ambiguity of the plan objectives and to provide a tool to compare plan results and it was adapted as a global measure of quality [32]. The previous PQI institutional definition for cervical cancer treatment has been used. The global index is defined as the quadratic mean of 4 sub-metrics considering the target coverage, the OAR sparing, the accuracy of plan delivery, and the plan complexity, each one of them ranging from 0 to 1 [21]. Each submetric score is increasing as the constraint fulfillment improves.

The results of the plan quality index and its sub-metrics are reported in Table 7 and Figure 6. The plan complexity sub-metric is not represented due to its very narrow distribution in each planning strategy which causes also the registered statistical significance in the variance analysis. The results revealed a statistically relevant decrease in the mCP02 coverage sub-metric. Finally, it is worth noticing the PQI trend of the mCP01 and mCP02 with respect to the gold standard MP: the first one slightly improves the overall plan quality, while the second one is almost comparable with the MP.

**Table 7** Comparison of original manual plans (MP) and mCycle plans (mCP01 and mCP02) in terms of Plan Quality Index (PQI) and its submetrics: coverage, OAR sparing, plan delivery accuracy, and plan complexity. Median values and ranges are reported.

| **PLAN QUALITY INDEX** |  |  | **mCP01** | | **mCP02** | |
| --- | --- | --- | --- | --- | --- | --- |
|  |  | **Median [range]** | **MVA**  **p-values** | **VVA**  **p-values** | **MVA**  **p-values** | **VVA**  **p-values** |
| Coverage ^(2)^ | MP | 0.90 [0.57 - 1.00] | **0.013**/0.195 | 0.722 | **<0.001/0.003** | 0.427 |
|  | mCP01 | 1.00 [0.30 - 1.00] |  |  | **<0.001/0.001** | 0.843 |
|  | mCP02 | 0.72 [0.15 - 0.86] |  |  |  |  |
|  |  |  |  |  |  |  |
| OAR sparing ^(2)^ | MP | 0.79 [0.32 - 1.00] | 0.507/1.000 | 0.135 | 0.150/1.000 | 0.343 |
|  | mCP01 | 0.81 [0.05 - 1.00] |  |  | 0.416/1.000 | 0.531 |
|  | mCP02 | 0.91 [0.50 - 1.00] |  |  |  |  |
|  |  |  |  |  |  |  |
| Plan delivery accuracy ^(2)^ | MP | 0.75 [0.30 - 1.00] | 0.466/1.000 | **0.002** | 0.858/1.000 | **0.005** |
|  | mCP01 | 0.80 [0.60 - 0.90] |  |  | 0.403/1.000 | 0.839 |
|  | mCP02 | 0.70 [0.50 - 0.90] |  |  |  |  |
|  |  |  |  |  |  |  |
| Plan complexity ^(2)^ | MP | 0.20 [0.20 - 0.30] | **0.004**/0.060 | **0.003** | **0.003/0.045** | **0.017** |
|  | mCP01 | 0.20 [0.20 - 0.20] |  |  | 0.342/1.000 | 0.324 |
|  | mCP02 | 0.20 [0.10 - 0.20] |  |  |  |  |
|  |  |  |  |  |  |  |
| PQI ^(2)^ | MP | 0.69 [0.41 - 0.84] | 0.159/1.000 | 0.302 | 0.560/1.000 | 0.869 |
|  | mCP01 | 0.73 [0.51 - 0.82] |  |  | 0.046/0.690 | 0.224 |
|  | mCP02 | 0.70 [0.48 - 0.78] |  |  |  |  |

Abbreviations: MVA: median value analysis, VVA: variance value analysis, OAR: organs at risk, ^(1)^: Gaussian distribution, ^(2)^: not normal distribution. Non-corrected and Bonferroni-corrected p-values are reported (p value/corrected p value). Bold: Statistical significance (p<0.05).

**
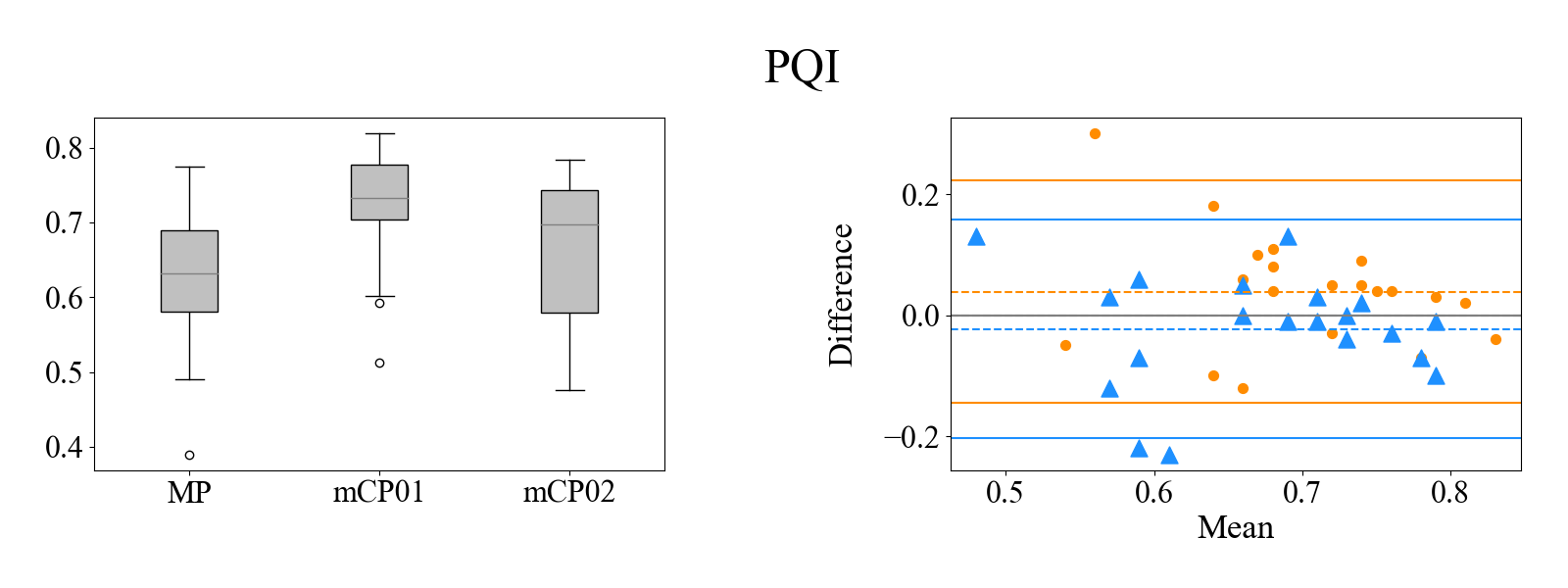
**

**Figure 6** Box-and-whisker plots (left) and related Bland Altman plots (right) for PQI for manual plans (MP) and mCycle plans (mCP01 and mCP02). In Bland Altman plots orange circles and blue triangles represent "MP vs mCP01" and “MP vs mCP02” comparison, respectively. Dashed lines: bias line, solid lines: agreement limits lines. Abbreviations: PQI: Plan Quality Index.
